# Association between neighborhood social cohesion, awareness of chronic diseases, and participation in healthy behaviors in a community cohort

**DOI:** 10.1186/s12889-021-11633-8

**Published:** 2021-09-03

**Authors:** Andrew M. Rosenblatt, Deidra C. Crews, Neil R. Powe, Alan B. Zonderman, Michele K. Evans, Delphine S. Tuot

**Affiliations:** 1 College of Arts and Sciences, Cornell University, Ithaca, NY USA; 2 Division of Nephrology, Department of Medicine, Johns Hopkins University School of Medicine, Baltimore, MD USA; 3 Center for Vulnerable Populations at Zuckerberg San Francisco General Hospital and Trauma Center, San Francisco, CA USA; 4 Department of Medicine, University of California, San Francisco, San Francisco, CA USA; 5 Laboratory of Epidemiology and Population Science National Institute on Aging, National Institutes of Health, Baltimore, MD USA; 6 Division of Nephrology, University of California, San Francisco, Zuckerberg San Francisco General Hospital and Trauma Center, 1001 Potrero Ave. Building 100, Room 342, San Francisco, CA 94110 USA

**Keywords:** Social cohesion, Kidney disease, CKD awareness, CKD, HANDLS, Healthy behaviors, Health disparities

## Abstract

**Background:**

Neighborhood social cohesion (NSC) is the network of relationships as well as the shared values and norms of residents in a neighborhood. Higher NSC has been associated with improved cardiovascular health, largely among Whites but not African Americans. In a bi-racial cohort, we aimed to study the association between NSC and chronic disease awareness and engagement in healthy self-management behaviors, two potential mechanisms by which NSC could impact cardiovascular health outcomes.

**Methods:**

Using the Healthy Aging in Neighborhoods of Diversity Across the Lifespan Study (HANDLS), we cross-sectionally examined the association between NSC and awareness of three chronic conditions (diabetes, chronic kidney disease (CKD), and hypertension) and engagement in healthy self-management behaviors including physical activity, healthy eating, and cigarette avoidance.

**Results:**

Study participants (*n* = 2082) had a mean age of 56.5 years; 38.7% were White and 61.4% African American. Of the participants, 26% had diabetes, 70% had hypertension and 20.2% had CKD. Mean NSC was 3.3 (SD = 0.80) on a scale of 1 (lowest score) to 5 (highest score). There was no significant association between NSC and any chronic disease awareness, overall or by race. However, each higher point in mean NSC score was associated with less cigarette use and healthier eating scores, among Whites (adjusted odds ratio [aOR], 95% confidence interval [CI]: =0.76, 0.61–0.94; beta coefficient [βc]:, 95% CI: 1.75; 0.55–2.97, respectively) but not African Americans (aOR = 0.95, 0.79–1.13; βc: 0.46, − 0.48–1.39, respectively; P_interaction_ = 0.08 and 0.06). Among both Whites and African Americans, higher NSC scores were associated with increases in self-reported physical activity (βc: 0.12; 0.08–0.16; P_interaction_ = 0.40).

**Conclusions:**

Community engagement and neighborhood social cohesion may be important targets for promotion of healthy behaviors and cardiovascular disease prevention. More research is needed to understand the different associations of NSC and healthy behaviors by race.

**Supplementary Information:**

The online version contains supplementary material available at 10.1186/s12889-021-11633-8.

## Background

Kidney disease, diabetes, and hypertension affect millions of people in the United States and are associated with cardiovascular morbidity and early mortality, particularly among African Americans compared to Whites [1–5]. Consequently, cardiovascular morbidity and early mortality could be decreased with greater individual awareness of chronic diseases and engagement in risk-lowering behaviors, such as glycemic control among individuals with diabetes and stopping tobacco use for all individuals with chronic disease [2, 6]. Nationally representative data suggest that participation in these behaviors is suboptimal [7], and large cohort studies have suggested differential engagement in healthy behaviors by socioeconomic status (SES), race, and educational attainment [8–11].

The U.S. Department of Health and Human Services has identified social cohesion as a major component to maintaining individual health [12], in part due to recent work examining the potential impact of neighborhood social cohesion (NSC) on individual health outcomes [13, 14]. NSC is the network of relationships, shared values, and norms of residents in a neighborhood and it shares some similarities to an individual’s social network [15]. It differs by accounting for value systems, degree of social interaction, and by considering the cohesion of a broader neighborhood rather than cohesion within a small group of individuals [16, 17].

NSC may affect the health of individuals residing close to one another through several mechanisms, such as the collective advocacy for resources, increased dissemination of health-related information, psychosocial support, and self-efficacy to engage in healthy behaviors. Recent studies have shown that stronger, denser social networks are correlated with goal attainment, general habit formation, and participation in cardiovascular risk reduction strategies [18, 19]. NSC may similarly serve as a catalyst for healthy behaviors by improving individuals’ perceived self-efficacy through the promotion of social and community ties [20], such as attendance at preventive healthcare visits, engagement in regular physical exercise, and tobacco cessation [21–24]. Personal awareness of disease or risk of developing disease is an upstream determinant to participation in healthy behaviors [25, 26] and might also be enhanced through strong social networks and neighborhood cohesion.

Prior U.S. studies have shown protective associations between higher NSC and some health outcomes, however results have been mixed and have differed by race. For example, data from the Chicago Health and Aging Project demonstrated that higher NSC was associated with decreased stroke mortality but not stroke incidence and was protective among Whites but not African Americans [27]. Data from the Multi-Ethic Study of Atherosclerosis suggested that higher NSC was associated with less hypertension but was not associated with kidney function decline; neither association differed by race/ethnicity [28, 29].

To explore the potential mechanism by which NSC could impact cardiovascular health events, we aimed to examine whether NSC was associated with awareness of three common chronic diseases that are risk factors for cardiovascular disease (chronic kidney disease, hypertension, diabetes) and engagement in healthy self-management behaviors. Since previous data suggested racial differences in the association between NSC and health outcomes, we examined these associations by race, leveraging a bi-racial cohort of socioeconomically diverse, community-dwelling adults.

## Methods

### Study design and participants

We conducted a cross-sectional study examining the relationship between individual perception of neighborhood social cohesion, awareness of health conditions, and participation in healthy behaviors among individuals who participated in the prospective longitudinal Healthy Aging in Neighborhoods of Diversity Across the Life Span (HANDLS) study, taking place between 2013 and 2017. To identify the interaction of race and socioeconomic status on the development of cardiovascular and cerebrovascular health disparities, the HANDLS study recruited community-dwelling African Americans and Whites aged 30–64 years from an area probability sample of 13 neighborhoods representing contiguous U.S. census tracts in Baltimore City. Based on 2000 census data, these neighborhoods were thought to yield sufficient individuals to recruit a minimum of 30 participants per cell defined by race (African American, White) socioeconomic status (above and below 125% of the 2004 Health and Human Services Poverty guidelines), age (seven 5-year age groups) and sex (male, female), necessary to detect a difference in cardiovascular outcomes with 80% power after 20 years. HANDLS participants were recruited in two phases: household recruitment and interview followed by examination in medical research vehicles belonging to the study. Detailed methods for recruitment in HANDLS have been reported elsewhere [30].

Overall, 3720 individuals participated in a Wave 1 (baseline) study visit between August 2004 and March 2009. Wave 4 visits occurred between September 2013 and September 2017 and consisted of in-person health examination, a telephonic dietary recall, renal function assessment, and optional participation in a telephone-based survey. Nearly 58% of Wave 1 participants (*n* = 2171/3720) had a Wave 4 visit, during which most data were collected for this ancillary study, including the primary predictor. This study population excluded individuals missing serum creatinine and urine albuminuria values (*n* = 71), and those with an estimated glomerular filtration rate (eGFR) < 15 ml/min/1.73m^2^ (*n* = 18) at Wave 4, for a final study population was 2082. National Institutes of Health Institutional Review Board (#09AGN248), approved the study protocol, as did the University of California, San Francisco Institutional Review Board (#10–02885). All participants provided written, informed consent to participate.

### Data collection and definitions

The primary explanatory variable was individual perception of neighborhood social cohesion, based on a validated questionnaire of 5 questions asking about unique neighborhood characteristics [17]. Individuals were asked to agree or disagree with the following statements using a 1–5 Likert scale in which 1 represented strong disagreement and 5 represented strong agreement: (1) “People in my neighborhood are willing to help their neighbors;” (2) “I live in a close-knit neighborhood;” (3) “People in my neighborhood can be trusted;” (4) “People in my neighborhood generally do not get along with each other;” and (5) People in my neighborhood do not share the same values.” Consistent with prior research, a composite neighborhood social cohesion score was calculated as the mean of the 5 items with items (4) and (5) reverse coded, with higher values representing more agreement that there was social cohesion [31, 32].

Awareness outcomes included awareness of kidney disease, diabetes, and hypertension, defined by accurate self-report of each of these chronic conditions during the Wave 4 health questionnaire compared to study measures of eGFR, fasting glucose/use of antidiabetic medications, and BP/use of anti-hypertensive medications [33]. Behavioral outcomes were defined by self-reported participation in healthy behaviors: no cigarette use, higher physical activity defined by the Baecke physical activity questionnaire [34], and heathy eating defined by the 2010 Healthy Eating Index [35].

Covariates included self-reported demographic information (age, sex, race, educational attainment, health insurance, having a regular healthcare provider, annual household income, and co-morbid conditions) obtained during Wave 1. Health literacy was measured with the hort Test of Functional Health Literacy in Adults (S-TOFHLA) using a cutoff score of 60 to differentiate between individuals with adequate vs. inadequate literacy levels [36]. Physical exam measures included blood pressure, height, and weight. Each participant underwent sitting and standing blood pressure measurements on each arm using the brachial artery auscultation method with an inflatable cuff of appropriate size. Laboratory measures included serum creatinine, urine microalbumin, serum glycated hemoglobin, and fasting glucose. Diabetes was defined by a fasting glucose > 126 ml/dl or use of an antidiabetic medication. Hypertension was defined by an uncontrolled measured BP defined by an average seated study systolic blood pressure > 140 mmHg or an average seated study diastolic BP > 90 mmHg or use of an anti-hypertensive medication. CKD was defined by single values of eGFR < 60 ml/min/1.73m^2^ calculated by the Chronic Kidney Disease Epidemiology Collaboration (CKD-EPI) equation or the presence a urine microalbumin-to-creatinine ratio (UACR) > 30 mg/g [37, 38].

### Statistical analysis

Characteristics of participants were compared by tertile of perceived social cohesion using χ^2^ and ANOVA tests as appropriate. Multivariable logistic regression models were used to determine the presence, direction, strength and independence of an association between mean social cohesion score (modeled as a continuous variable) and awareness of each chronic condition (CKD, diabetes, hypertension) and cigarette use (all binary variables). Multivariable linear regression models were used to determine the association of mean social cohesion score with physical activity and healthy eating scores modeled as continuous variables. All models represented complete case analyses and included variables that were either determined a priori based on prior literature or statistically associated with tertile of social cohesion. Model 1 included age, sex, and race. Model 2 further adjusted for additional demographic variables (educational attainment, health insurance status, and having a regular healthcare provider). Model 3 further adjusted for clinical variables (diabetes status, hypertension status, eGFR, and UACR) when appropriate (i.e., models examining diabetes awareness did not include diabetes status; models examining hypertension awareness did not include hypertension status). Interaction between race and neighborhood social cohesion was assessed in each fully adjusted model. All statistical analyses were performed using STATA software, Version 14.2 (StataCorp, College Station, Texas, USA).

## Results

### Cohort characteristics

Among the study population of 2082 individuals, mean (SD) age was 56.5 (9.1) years, 40.9% were men, 38.7% were White, and 61.4% were African American. Mean NSC for the entire cohort was 3.3 (SD = 0.80) with a mean score in the first NSC tertile of 2.55 (standard deviation [SD] = 0.51), a mean score of 3.40 (SD = 0.16) in the second NSC tertile, and a mean score of 4.23 (SD = 0.38) in the third NSC tertile. Among the entire cohort, 32.3% did not complete their high school education and 12.7% had low health literacy, percentages that did not differ by social cohesion tertile. Approximately 39% of the cohort lived below the 125% federal poverty level with fewer individuals living below the poverty level among those who self-reported higher social cohesion (*p* < 0.001). Nearly 65% of participants reported having health insurance and 68.8% cited a regular source of health care, with higher percentages of individuals reporting either characteristic with increasing social cohesion score (*p* = 0.002 and *p* = 0.04, respectively). Of the overall cohort, 26.3% were diabetic and 70% had hypertension. Approximately 58.9% of individuals had CKD stage 3 or 4 and 13.9% had urine albuminuria levels > 30 mg/g of creatinine (Table 1).

**Table 1:** Characteristics of the study population, by tertile of social cohesion

Characteristics	All^a^	Tertile 1 Social Cohesion Score	Tertile 2 Social Cohesion Score	Tertile 3 Social Cohesion score	***P*** Value
		range: 1.-0–3.0	range: 3.2–3.6	range: 3.7–5.0	
	*N* = 2082	***N*** = 792	***N*** = 565	***N*** = 561	
Overall Mean NSC (SD)	3.29 (0.80)	2.55 (0.51)	3.39 (0.16)	4.24 (0.38)	
Age, mean (SD)	56.5 (9.1)	55.8 (9.0)	56.9 (9.0)	56.9 (9.4)	0.03
Female, N (%)	1133 (59.1)	492 (62.1)	300 (53.1)	341 (60.8)	0.002
Race/ethnicity, N (%)					< 0.001
White	792 (38.7)	325 (41.0)	183 (32.3)	241 (43.0)	
African American	1278 (61.4)	467 (59.0)	382 (67.6)	320 (57.0)	
Educational attainment, N (%)					< 0.001
< High school	604 (32.3)	283 (36.7)	197 (35.6)	124 (22.6)	
High school graduate	1023 (54.6)	418 (54.2)	300 (54.3)	305 (55.7)	
College graduate	245 (13.1)	70 (9.1)	56 (10.1)	119 (21.7)	
Poverty (< 125% poverty level), N (%)	755 (39.4)	356 (45.0)	228 (40.4)	171 (30.5)	< 0.001
Low health literacy, N (%)	264 (12.7)	99 (17.2)	79 (18.3)	57 (12.7)	0.05
Has health insurance, N (%)	1351 (64.9)	490 (61.9)	359 (63.5)	398 (70.9)	0.002
Regular source of healthcare, N (%)	1290 (68.8)	477 (61.7)	343 (62.0)	373 (68.0)	0.04
Diagnosed diabetes, N (%)	504 (26.3)	216 (27.3)	121 (21.4)	114 (20.4)	0.005
Diagnosed hypertension, N (%)	1340 (70.0)	510 (64.4)	371 (65.8)	344 (61.5)	0.32
BMI, mean (SD)	30.9 (7.9)	31.2 (8.1)	30.6 (7.2)	30.7 (7.9)	0.36
Hemoglobin A1c, mean (SD)	6.2 (1.3)	6.2 (1.3)	6.2 (1.2)	6.1 (1.3)	0.36
SBP (mmHg), mean (SD)	117.3 (20.3)	117.4 (19.8)	117.7 (19.3)	116.3 (21.8)	0.39
DBP (mmHg), mean (SD)	65.6 (11.1)	65.7 (10.6)	65.8 (10.4)	64.7 (12.2)	0.15
eGFR (ml/min/1.73m^2^), mean (SD)	80.8 (18.5)	81.0 (18.1)	80.4 (18.3)	81.4 (18.7)	0.62
eGFR < 60 min/min/1.73m^2^, N (%)	271 (58.9)	113 (61.4)	85 (58.6)	73 (55.7)	0.39
Albuminuria (mg/g), median (IQR)	5.5 (3.5, 12.8)	5.5 (3.5, 12.6)	5.4 (3.5, 13.9)	5.1 (3.2, 11.3)	0.42
Albuminuria > 30 mg/g, N (%)	263 (13.9)	110 (14.1)	83 (14.8)	70 (12.7)	0.59

^a^Among the entire cohort, *n* = 2082 for all rows except: Age: (*n* = 1917), Educational attainment (total *n* = 2034), Health literacy (total *n* = 1558), Regular source of healthcare (total *n* = 2036); Diagnosed hypertension (total *n* = 207), Diagnosed diabetes (total *n* = 2081), BMI (*n* = 1903), Hemoglobin A1c (total *n* = 1893), SBP and DBP (total *n* = 1910), eGFR (*n* = 1904), albuminuria total (*n* = 1893). Approximately 91.7% of the population (*n* = 1918) answered questions about social cohesion

Wave 4 participants were generally similar to those who completed the baseline study visit. However, Wave 4 participants were more likely to be female compared to those participants who were lost to follow-up (58.6% vs. 49.2%, *p* < 0.01) and more likely to be African American (61.1% vs. 56.3%, *p* = 0.003). Education, poverty status and insurance were not different among the two groups (data not shown, *p* > 0.05). Of the Wave 4 study population, 1918 individuals answered questions about social cohesion. While individuals who did not complete the NSC questionnaire were generally similar to those who did, individuals missing NSC scores were less likely to be a college graduate, more likely to have low health literacy, and more likely to have diagnosed hypertension and diabetes. (Supplemental Table).

### Neighborhood social cohesion and awareness of chronic disease

**Fig. 1:**
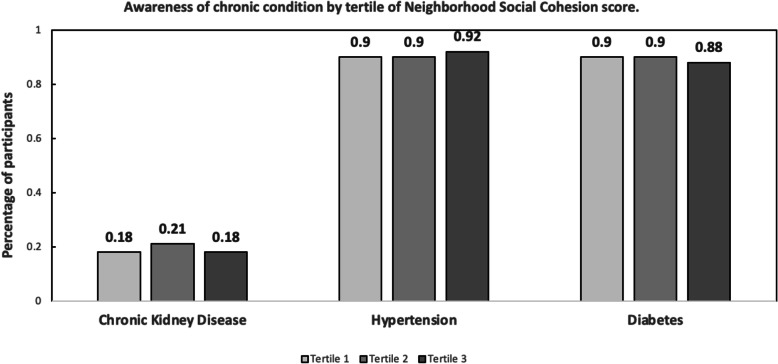
Participant awareness of chronic disease by tertile of neighborhood social cohesion score

Overall, 18.7% (86/460) of individuals with CKD were aware of their disease. Among those aware of their CKD, mean NSC score was 3.32 (SD = 0.73) with similar prevalence of awareness by tertile of neighborhood social cohesion (Fig. 1). Nearly 90% of individuals with diabetes (89.7%, 330/368) were aware of their condition. Of those aware, mean NSC score was 3.20 (SD = 0.78). A similar, high percentage of individuals (90.7%, *n* = 936/1032) reported awareness of hypertension with a similar distribution of awareness by neighborhood social cohesion scores; mean NSC among those aware was 3.28 (SD = 0.79).

Using multivariable logistic regression, increasing mean social cohesion score was not significantly associated with awareness of CKD (adjusted odds ratio [aOR] = 1.13; 95% Confidence Interval = 0.81, 1.56), awareness of diabetes (aOR = 0.94; 0.57–1.55) or awareness of hypertension (aOR = 1.15; 0.87–1.51). None of the associations between NSC and awareness differed by race (P_interaction NSC x race_ > 0.10).

### Neighborhood social cohesion and healthy behaviors

Neighborhood social cohesion scores were independently associated with many healthy lifestyle habits and often differed by race (Table 2). For example, there were 13.1% decreased odds of cigarette use with every one-point gain in self-reported mean social cohesion score (aOR = 0.87; 0.76, 0.99), an association largely driven among White participants (P_interaction NSC x race_ = 0.08). Among Whites, odds of cigarette use were 24% lower with each higher point in NSC (aOR = 0.76; 0.61–0.94) while there was no association between cigarette use and NSC score among African Americans (aOR = 0.95; 0.79–1.13). Similarly, every one-point increase in mean NSC score was associated with an increase in healthy eating engagement according to the Healthy Eating Index, (beta coefficient [βc]: 1.08; 95% Confidence Interval = 0.34–1.82), largely driven by White participants (P_interaction NSC x race_ = 0.06). Among Whites, every one-point increase in mean NSC was associated with an increase of healthy eating engagement score (βc: 1.75; 0.55–2.97) while the association was not statistically significant among African Americans (βc: 0.46; − 0.48–1.39). Higher neighborhood social cohesion scores were associated with increased physical activity (βc: 0.12; 0.08–0.16) without any difference by race (P_interaction NSC x race_ = 0.40).

**Table 2:** Characteristics associated with healthy behaviors (cigarette use, healthy eating, physical activity), stratified by race. Adjusted odds ratios (aOR) and 95% Confidence Intervals (CI) are presented for the logistic model; beta coefficients (βc) and 95% CI are presented for linear models

Characteristics	Cigarette use		Healthy eating		Physical activity	
	African Americans aOR (95% CI)	Whites aOR (95% CI)	African Americans βc (95% CI)	Whites βc (95% CI)	African Americans βc (95% CI)	Whites βc (95% CI)
Mean neighborhood social cohesion score	0.95 (0.80, 1.14)	**0.76 (0.61, 0.94)**	0.46 (−0.48, 1.39)	**1.75 (0.53, 2.97)**	**0.09 (0.04, 0.15)**	**0.14 (0.08, 0.21)**
Age (per year)	**0.97 (0.95, 0.98)**	**0.98 (0.95, 0.99)**	**0.28 (0.19, 0.37)**	**0.15 (0.01, 0.28)**	**−0.07 (− 0.01, − 0.002)**	**−0.003 (− 0.01, 0.004)**
Female sex	**0.67 (0.51, 0.89)**	1.24 (0.87, 1.78)	0.59 (−0.85, 2.05)	**2.71 (0.62, 4.80)**	**−0.12 (− 0.19, − 0.04)**	**−0.17 (− 0.28, − 0.05)**
High school graduate or above	**0.62 (0.47, 0.84)**	**1.54 (0.37, 0.78)**	**2.78 (1.24, 4.32)**	**4.13 (1.86, 6.39)**	**0.07 (−0.01, 0.16)**	**0.16 (0.04, 0.29)**
Poverty (< 125% federal poverty level)	**1.53 (1.17, 2.02)**	**1.45 (0.99, 2.01)**	−1.88 (−3.31, −0.44)	**−3.44 (−5.69, − 1.18)**	−0.03 (− 0.11, 0.05)	−0.17 (− 0.29, − 0.04)
Insurance status	0.82 (0.59, 1.13)	0.82 (0.52, 1.26)	1.49 (− 0.27, 3.26)	1.67 (− 1.03, 4.37)	−0.06 (− 0.16, 0.04)	−0.03 (− 0.18, 0.12)
Regular provider for healthcare	**0.72 (0.52, 1.00)**	0.55 (0.36, 0.85)	1.19 (0.87, 1.61)	1.99 (−0.68, 4.66)	−0.003 (− 0.10, 0.09)	**0.12 (− 0.03, 0.27)**
Diabetes diagnosis	**0.70 (0.51, 0.98)**	1.13 (0.72, 1.76)	1.37 (−0.31, 3.05)	2.14 (− 0.47, 4.74)	**−0.17 (− 0.26, − 0.08)**	**−0.25 (− 0.39, − 0.10)**
Hypertension diagnosis	1.09 (0.80, 1.49)	0.92 (0.63, 1.34)	− 0.80 (−2.44, 0.83)	−1.49 (− 3.69, 0.71)	**−0.17 (− 0.26, − 0.08)**	**−0.26 (− 0.38, − 0.14)**
eGFR (per ml/min/1.73m^2^)	1.00 (1.00, 1.01)	1.01 (0.99, 1.02)	**0.04 (− 0.006, 0.08)**	−0.02 (− 0.09, 0.05)	0.0009 (− 0.001, 0.003)	0.005 (0.001, 0.009)
uACR (per mg/g)	0.99 (0.99, 1.00)	1.00 (1.00, 1.00)	−0.0005 (− 0.004, 0.003)	−0.0005 (− 0.004, 0.003)	−6 × 10^− 5^ (− 0.0002, 0.00008)	−2.27 × 10^− 6^ (− 0.0001, 0.0001)

Models are adjusted for all covariates listed

*eGFR* estimated glomerular filtration rate, *UACR* urine albumin:creatinine ratio

Other sociodemographic characteristics were also independently associated with participation in healthy behaviors. As depicted in Table 2, younger age was associated with higher odds of cigarette use (aOR = 0.97; 0.96–0.98) and less physical activity (βc: − 0.005; − 0.01–0.001) whereas older age was associated with higher report of healthy eating habits (βc: 0.23; 0.12–0.31). Having a greater than high school education was uniformly helpful, associated with lesser odds of cigarette use (aOR = 0.58; 0.47–0.73), greater healthy eating (βc: 3.23; 1.95–4.51), and more physical activity (βc: 0.11; 0.04–0.19). Poverty, on the other hand, was associated with greater odds of cigarette use (aOR = 1.51; 1.21–1.87) and non-statistically associated with less healthy eating (βc: − 2.45; − 3.68 – − 1.23) and less physical activity (βc: − 0.08; − 0.15–0.01). These values are stratified by race in Table 2.

## Discussion

We examined the relationship between NSC and two potential mechanisms by which it could affect cardiovascular outcomes: awareness of chronic disease and engagement in healthy self-management behaviors. We found no association between NSC and individual awareness of chronic disease, overall or by race, which is a novel contribution to the literature on this topic. Consistent with data from prior studies, we found that higher neighborhood social cohesion scores were associated with greater engagement in healthy self-management behaviors among Whites but less so among African Americans.

Successful habit formation is aided by the presence of a significant social support network that can encourage adherence to or abstinence from a habit or behavior whereby individuals may observe the consequences of engaging or not engaging in a behavior by their peers [39]. According to social cognitive theory, individuals with high self-efficacy expectancies—the belief in one’s capacity to achieve one’s goals—are healthier, more effective at engaging in healthy lifestyles, and generally more successful than those with low self-efficacy expectancies [40]. High self-efficacy can be developed through a support network of trusted or respected peers [20]. If higher levels of neighborhood social cohesion provide a stronger social network, then social cognitive theory suggests that an individual would be likelier to engage in healthy behaviors in a highly socially cohesive environment [41]. This would allow individuals to collectively advocate for resources and knowledge, potentially increase individual self-efficacy, and have greater exposure to the positive and negative consequences of engaging or disregarding healthy self-management behaviors.

Our results suggesting that higher NSC is associated with higher individual engagement in self-management behaviors is consistent with prior work demonstrating greater receipt of preventive health measures, such as the influenza vaccine, cholesterol testing, and age appropriate cancer screening with higher reported NSC [24, 41, 42]. These results have implications for the encouragement of behaviors which may prevent chronic disease onset or progression. In addition to the traditional channel of encouraging healthy self-management behaviors through a primary care provider and the primary care medical home, medical professionals and community leaders could improve public health by sponsoring and organizing social community engagement initiatives which increase the social cohesion of the neighborhood, even if they are not directly related to health, such as neighborhood walks and park cleanups [43]. These gatherings could also incorporate more traditional public health initiatives such as disease-screening fairs and healthy cooking workshops. Many efficacious health-related community initiatives have enhanced disease detection and awareness [44–47], but none to our knowledge have been evaluated for their impact on social cohesion, providing another method to examine the public health impact of such programs.

The differences in association between NSC and tobacco use and healthy eating by race have been shown previously [48, 49], and is consistent with known racial differences in cardiovascular outcomes but merits further investigation. One possible explanation is that social cohesion can be a detriment as well as a benefit. While we did not have data on neighborhood composition, prior work suggested that Baltimore’s neighborhoods have a high degree of social racial segregation [50]. If study participants’ neighborhoods and social circles were relatively homogenous in their racial composition and healthy behavior engagement was higher among Whites compared to African Americans, increased social cohesion would promote healthier behaviors among one group and not the other. This is consistent with known African American-White inequalities in hypertension, diabetes, and obesity in areas with more racial segregation [10, 51]. Similarly, it may also be that Whites had greater access to resources that would allow them to choose healthier diets and to quit smoking if encouraged by their strong neighborhood ties, while African Americans may have wanted to make the same choices when encouraged but could not realize them. However, we did not see these differences extended to physical activity, which would be expected. Future work should examine the role of racial segregation among residential neighborhoods with engagement in healthy self-management behaviors.

We did not find an association between NSC and participants’ awareness of their own chronic disease. We thought that awareness of disease might be an inciting factor in behavior change and that social cohesion might work through this mechanism. Active participation in healthy self-management behaviors is likely more impacted by self-efficacy and social networks than knowledge about one’s own disease [52, 53]. This hypothesis could explain why other studies have found lower prevalence of hypertension [54] and type 2 diabetes [55] in neighborhoods with high social cohesion, meriting further investigation of the interaction between social cohesion and chronic disease.

Our findings must be appreciated within the limitations of this study. First, causality cannot be inferred because of the cross-sectional nature of this study. Also, reverse causality (i.e. higher participation in healthy behaviors leading to increased self-reported NSC) cannot be ruled out, and there may be residual confounding, as with all observational studies. Second, CKD status was defined by single measurements of eGFR or albuminuria, potentially leading to misclassification. Third, participation in healthy behaviors was self-reported which may have introduced subjectivity to the reports, potentially through social desirability bias, which may differ by race, though definitive data are lacking [56]. These inaccuracies could also be differential with respect to NSC, in that social biases may have a stronger effect where there is greater NSC. Finally, we lacked data on the racial composition of individual neighborhoods in this study, so results may not be generalizable to non-urban populations across the United States. Selection bias resulting from differences in who agrees to participate and remain in a longitudinal cohort study may limit generalizability.

## Conlcusions

In summary, our results show a significant association between higher NSC scores and increased participation in healthy self-management behaviors, particularly among Whites, but no association between NSC and disease awareness. These findings suggest that individual engagement in healthy behaviors may be a mediator in the association between high NSC and fewer adverse health outcomes for individuals residing in neighborhoods where healthy behaviors are widely practiced. While these racial differences merit greater study, our results indicate that targeting community engagement and bolstering the social cohesion of a neighborhood could have significant benefits for communities’ public health.

## Supplementary Information

**Additional file 1: Supplemental Table**. Characteristics of the study population, by missingness of NSC.

## Data Availability

HANDLS data and code may be made available from the corresponding author upon request.

## Abbreviations

NSC: Neighborhood social cohesion
CKD: Chronic kidney disease
SES: Socioeconomic status
HANDLS: Healthy Aging in Neighborhoods of Diversity Across the Life Span
eGFR: estimated glomerular filtration rate
TOFHLA: Test of Functional Health Literacy in Adults
BP: Blood pressure
CKD-EPI: Chronic Kidney Disease Epidemiology Collaboration
UACR: Urine albumin-to-creatinine ratio
aOR: Ajusted odds ratio
