# Supplementary material for: Association between neighborhood social cohesion, awareness of chronic diseases, and participation in healthy behaviors in a community cohort
Source: BMC Public Health. 2021 Sep 3;21:1611. doi: 10.1186/s12889-021-11633-8 (PMC8414876; doi:10.1186/s12889-021-11633-8)
Supplement: Supplementary file 1 — Additional file 1: Supplemental Table. Characteristics of the study population, by missingness of NSC. [file 12889_2021_11633_MOESM1_ESM.docx]

| Supplemental Table. Characteristics of the study population, by missingness of NSC. | | | |
| --- | --- | --- | --- |
| **Characteristics** | **NSC answered** | **NSC missing** | **P Value** |
|  |  |  |  |
|  | **N=1997** | **N=1723** |  |
| Age, mean (SD) | 56.5 (9.15) | 57.5 (8.63) | 0.17 |
| Female, N (%) | 1179 (59.04) | 94 (54.02) | 0.20 |
| Race/ethnicity, N (%) |  |  | 0.06 |
| White | 789 (39.51) | 733 (40.91) |  |
| African American | 2108 (60.49) | 990 (57.46) |  |
| Educational attainment, N (%) |  |  | 0.002 |
| < High school | 673 (34.53) | 656 (39.16) |  |
| High school graduate | 1063 (54.54) | 881 (52.60) |  |
| College graduate | 213 (10.93) | 138 (8.24) |  |
| Poverty (<125% poverty level), N (%) | 795 (39.81) | 740 (42.95) | 0.05 |
| Low health literacy, N (%) | 238 (15.87) | 29 (27.88) | 0.001 |
| Has health insurance, N (%) | 1306 (66.94) | 1076 (64.28) | 0.09 |
| Regular source of healthcare, N (%) | 1248 (63.97) | 104 (60.47) | 0.36 |
| Diagnosed diabetes, N (%) | 473 (23.72) | 55 (32.74) | 0.009 |
| Diagnosed hypertension, N (%) | 1278 (64.12) | 118 (73.75) | 0.01 |
| BMI, mean (SD) | 30.9 (7.87) | 30.8 (8.88) | 0.89 |
| Hemoglobin A1c, mean (SD) | 6.18 (1.27) | 6.55 (2.02) | 0.0007 |
| SBP (mmHg), mean (SD) | 116.9 (20.59) | 119.8 (21.69) | 0.10 |
| DBP (mmHg), mean (SD) | 65.5 (11.49) | 67.0 (11.65) | 0.11 |
| eGFR (ml/min/1.73m^2^), mean (SD) | 80.4 (19.35) | 77.9 (22.02) | 0.11 |
| Albuminuria (mg/g), median (IQR) | 51.7 (3.45, 12.69) | 65.0 (3.83, 18.18) | 0.59 |
